# Microbial Communities in Standing Dead Trees in Ghost Forests are Largely Aerobic, Saprophytic, and Methanotrophic

**DOI:** 10.1007/s00284-024-03767-w

**Published:** 2024-06-19

**Authors:** Mary Jane Carmichael, Melinda Martinez, Suzanna L. Bräuer, Marcelo Ardón

**Affiliations:** 1 Departments of Biology and Environmental Studies, Hollins University, Roanoke, VA 24020 USA; 2 U.S. Geological Survey, Eastern Ecological Science Center, Laurel, MD 20708 USA; 3 Department of Biology, Appalachian State University, Boone, NC 28608 USA; 4 Department of Forestry and Environmental Resources, North Carolina State University, Raleigh, NC 27695 USA

## Abstract

Standing dead trees (snags) are recognized for their influence on methane (CH_4_) cycling in coastal wetlands, yet the biogeochemical processes that control the magnitude and direction of fluxes across the snag-atmosphere interface are not fully elucidated. Herein, we analyzed microbial communities and fluxes at one height from ten snags in a ghost forest wetland. Snag-atmosphere CH_4_ fluxes were highly variable (− 0.11–0.51 mg CH_4_ m^−2^ h^−1^). CH_4_ production was measured in three out of ten snags; whereas, CH_4_ consumption was measured in two out of ten snags. Potential CH_4_ production and oxidation in one core from each snag was assayed in vitro. A single core produced CH_4_ under anoxic and oxic conditions, at measured rates of 0.7 and 0.6 ng CH_4_ g^−1^ h^−1^, respectively. Four cores oxidized CH_4_ under oxic conditions, with an average rate of − 1.13 ± 0.31 ng CH_4_ g^−1^ h^−1^. Illumina sequencing of the V3/V4 region of the 16S rRNA gene sequence revealed diverse microbial communities and indicated oxidative decomposition of deadwood. Methanogens were present in 20% of the snags, with a mean relative abundance of < 0.0001%. Methanotrophs were identified in all snags, with a mean relative abundance of 2% and represented the sole CH_4_-cycling communities in 80% of the snags. These data indicate potential for microbial attenuation of CH_4_ emissions across the snag-atmosphere interface in ghost forests. A better understanding of the environmental drivers of snag-associated microbial communities is necessary to forecast the response of CH_4_ cycling in coastal ghost forest wetlands to a shifting coastal landscape.

**Supplementary Information:**

The online version contains supplementary material available at 10.1007/s00284-024-03767-w.

## Introduction

The spatial footprint of ghost forests is expanding regionally and globally as a result of surface-(i.e., sea level rise) and subsurface (i.e., saltwater intrusion)-based salinization of coastal freshwater ecosystems [1–3]. As forest retreat rates are accelerating [1], Kirwan and Gedan predict that saltwater-driven land conversion will lead to broad-scale changes in coastal ecosystem structure and function in the coming years. Coastal forested wetlands provide feedback mechanisms to climate through the balance of carbon sequestration and emissions of greenhouse gases, particularly CH_4_ [4]. Therefore, a better understanding of how the conversion of coastal forested wetlands from live to ghost forests will be necessary to predict how climate-change associated stressors will influence carbon dynamics in these systems.

While the physical and biogeochemical processes that influence deadwood decomposition in upland systems has been well studied (e.g., [5–8]), much less is known about factors that influence deadwood decomposition in lowland systems, where sediment anoxia is more prevalent and deadwood is more prone to water saturation [9]. Differences in physical and biogeochemical parameters between upland and lowland systems, particularly as they relate to the presence and prevalence of oxic and anoxic microenvironments, may influence microbial community structure and, thus, the balance of aerobic vs. anaerobic decomposition pathways in deadwood. A product of the anerobic degradation of carbon compounds is CH_4_, a powerful greenhouse gas with a global warming potential ca. 28–36× that of CO_2_ over a 100-year time period [10]. As wetlands are the single largest source of global CH_4_ emissions [11], an improved understanding of factors that influence dominant decomposition pathways, and thus CH_4_ emissions, from forested wetland systems is important.

Methane dynamics in wetlands are controlled by microbial communities, more specifically the balance between methanogenesis and methanotrophy. Methanogenesis is the terminal step in the anaerobic degradation of carbon, which occurs in nutrient-depleted, anoxic microsites [12, 13]. The majority of CH_4_ production in wetlands is mediated by methanogenic archaea [14]. Recent evidence indicates that both fungi [15] and cyanobacteria [16] have the capacity to produce CH_4_ in aerobic conditions, though the quantitative importance of these taxa in methane production remains unresolved. Methane produced in wetlands has a variety of fates including: escape to the atmosphere via sediment-[17, 18], water-[19, 20], or plant-atmosphere interface [21–23] and consumption by methanotrophic bacteria and archaea (Table S1 and citations therein) that are widely distributed in the environment [24–26].

Approximately 15–30% of estimated total global wetlands are forested [27, 28], and woody vegetation has been shown to be a pathway for CH_4_ flux [23, 29]. Research showing the importance of living trees in CH_4_ emissions from Amazon floodplain forests [30] promoted interest on the role of plants in CH_4_ exchange. Covey and Megonigal [31] provide a comprehensive review of plant CH_4_ exchange in trees and forests, suggesting that CH_4_ fluxes through live trees might be an important and understudied pathway. The trunks of snags have also been recognized as both a source and sink of methane emissions in lowland coastal systems [21, 32, 33]. Much of the prior literature on deadwood carbon dynamics focused on coarse woody debris and felled trees in upland forests [34, 35], thus there is a need to more fully understand processes that affect deadwood decomposition in lowland systems, particularly in deadwood stocks that are standing or suspended [36, 37]. As a result of this recent expansion of research on live and dead trees, CH_4_ emissions from tree stems have been identified as *a new frontier in the global carbon cycle* [38], with calls for more research to better elucidate the biophysical mechanisms that modulate CH_4_ flux across this understudied atmospheric interface.

Both methanogens [39–41] and methanotrophs [42–44] have been identified in the phyllosphere (i.e., the above-ground parts of a plant [45]). The relative balance between these two functional guilds has the potential to drive plant CH_4_ exchange. Microbial communities in live trees have been shown to modulate plant CH_4_ exchange from lowland systems, reducing CH_4_ emissions across the plant-atmosphere interface by 30–40% [43, 46]. However, there is limited knowledge on the role of phyllosphere-associated microbial communities in modulating carbon fluxes from snags in lowland systems, despite isotopic evidence that microbial oxidation of CH_4_ occurs within the trunks of snags [47].

In this study, we used high-throughput Illumina sequencing of the V3/V4 region of the 16S rRNA gene to survey the microbial community in the trunks of ten snags in a ghost forest wetland at Gull Rock State Game Lands in Hyde County, North Carolina. Static flux chambers and snag core incubations were used to assess CH_4_ dynamics across the plant-atmosphere interface. A better understanding of the presence, prevalence, and activity of methanogens and methanotrophs in these phyllosphere-associated communities can support our ability to more accurately forecast the response of CH_4_ dynamics in forested wetland ecosystems to a shifting coastal landscape [34, 39, 48].

## Materials and Methods

### Site Description and Snag Selection

**Fig. 1:**
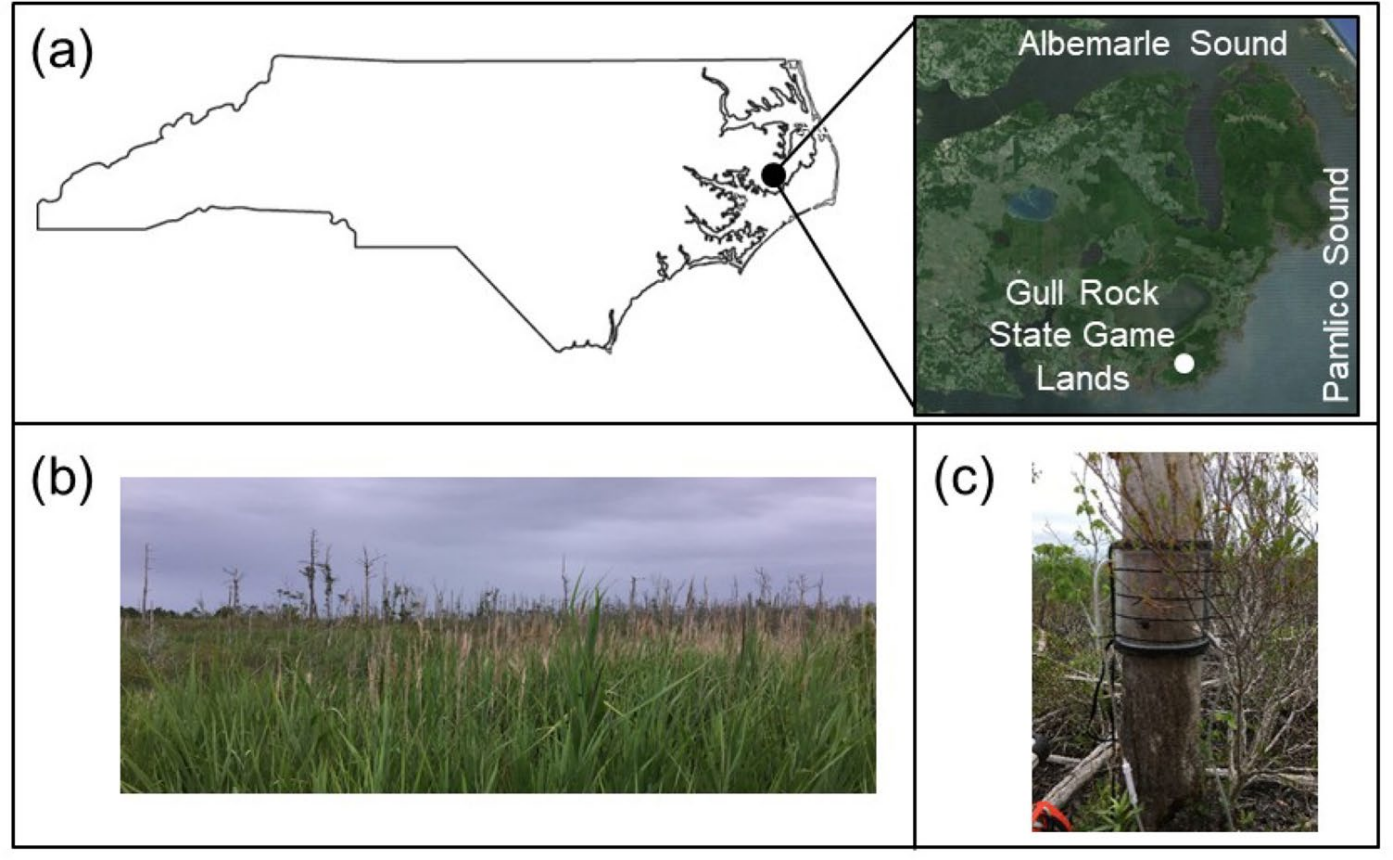
Location of study site in Hyde County, North Carolina, USA: (**a**) Location of Gull Rock State Game Lands in relation to the state of North Carolina and the Albemarle-Pamlico Peninsula, (**b**) ghost forest landscape where standing dead trees were sampled, (**c**) representative snag with static chamber and Gasmet DX4040 tubing attached. Panel (**a**) was created using Google Earth; image is copyrighted by DigitalGlobe (2016)

The Albemarle Pamlico Peninsula (Fig. 1a), located in eastern North Carolina, is surrounded by two brackish bodies of water: the Albemarle Sound to the north and the Pamlico Sound to the east and south [49]. Fifty percent of the peninsula is within 1.5 m of mean sea-level [50]. Due to the potential for high productivity, much of the peninsula was converted into croplands in the late 1970s [51]. In order to maintain aerated soils, an artificial drainage network was installed. The low-lying elevation of the peninsula [49] and the extensive drainage infrastructure makes the Albemarle Pamlico peninsula vulnerable to saltwater intrusion [50, 52, 53]. As a result, ghost forests have expanded in coastal North Carolina over the past few decades [2].

Our study took place in the growing season during June 2019 at Gull Rock State Game Lands (Fig. 1a), a 28,400 acre preserve in Hyde County, North Carolina that is more extensively described in Martinez and Ardón and Martinez et al. [33, 47]. The ghost forest where samples were taken (Fig. 1b) is located on the access road to the E. Merle Edwards/Loop Road Impoundment. The snags at the site were all pines, most likely *Pinus serotina* Michx., in various stages of decay. Snags were systematically selected (n = 10) to ensure that they were structurally sound enough to support static flux chambers and withstand drilling [21]. The mean height and diameter at breast height (DBH) of the snags were 8.3 ± 4.5 m (range 2.5–15.1 m) and 0.26 ± 0.09 m (range 0.12–0.38 m), respectively. A detailed description of the vegetation at the site and how it has changed over recent decades in response to saltwater intrusion can be found in Taillie et al. [54].

### Snag-Atmosphere Methane Fluxes

We used static chambers [55] to measure snag-atmosphere CH_4_ fluxes on all ten snags at heights ranging from 60–120 cm. Semi-rigid, vented chambers were designed after Siegenthaler et al. [56] and temporarily installed on snags in a location on each trunk that allowed for optimal sealing of the chamber [33]. Snag-atmosphere CH_4_ fluxes were measured using two methods, as described previously [33, 47]. A Gasmet DX4040 portable gas analyzer (Gasmet Technologies, Finland) was used to quantify CH_4_ flux over a 15 min interval, with measurements taken continuously every 20 s over the duration of the incubation and validated using an Agilent 7890A gas chromatograph (Agilent Systems, Santa Clara, CA) as described previously [33]. Linear regression models were used to calculate CH_4_ fluxes. An incubation met the assumption of linearity when r^2^ > 0.85 [21] and was considered significantly different from 0 when P < 0.05.

### Methane Sampling from Trunk Airspace

To confirm the presence of CH_4_ in trunk airspace, a protocol inspired by Covey et al. [57] and described in detail by Carmichael et al. [21] and Martinez et al. [47] was utilized on the ten snags selected for sampling. Sampling occurred immediately after the static flux chambers were removed from the snags. Three holes (30 cm, 60 cm, and 120 cm above ground level) were drilled horizontally from the bark to the center of the trunk using a 1.27 cm drill bit. Immediately after drilling, each hole was plugged with a 15 mm SubaSeal stopper (Sigma-Aldrich, St. Louis, MO), which was then pierced with a sampling wand connected to the Gasmet for 5 min. Air temperature, wind speed, and barometric pressure were noted at 0 and 5 min to ensure there were no major fluctuations over the duration of the sampling period. In addition, ambient air immediately outside of each drilled hole was sampled for 2 min after each trunk sample for comparison and to flush the trunk gases out of the Gasmet system before each subsequent sample.

For gas samples obtained from trunk airspace, t-tests were used to evaluate measured differences in CH_4_ concentrations between trunk airspace and ambient air next to the trunk at each measured height. A one-way analysis of variance was used to evaluate the comparison between CH_4_ concentrations in trunk airspace and ambient air next to the trunk at all heights above water level. A Shapiro–Wilk test for normality was run on each statistical analysis. If normality assumptions were violated, a non-parametric equivalent of the test (i.e., a Mann–Whitney Rank Sum test or Kruskal–Wallis one-way ANOVA on ranks with a Tukey test for multiple comparisons) was used to test for significance (P < 0.05). All statistical analyses were conducted using Sigma Plot v.14.5 (Systat Software, Palo Alto, CA).

### Potential CH_4_ Production and Oxidation Incubations

After CH_4_ sampling from trunk airspace was completed, duplicate cores were taken from each snag at 60 cm height to measure potential methane production (PMP) and potential methane oxidation (PMO) following Chan and Parkin [58]. Cores were extruded and sealed in a plastic straw that was duct taped on both ends, then stored on ice until receipt in the lab. One core was used for PMP assays, and the second was used to measure PMO as described in detail in Martinez et al. [47]. PMP was measured under anaerobic conditions, using ultra high purity (UHP) N_2_ as a headspace gas. PMO was measured under aerobic conditions, with the headspace of vials spiked above ambient CH_4_ to ca. 45 ppm using UHP CH_4_. Snag core CH_4_ fluxes were calculated as described in Martinez et al. [47]. The sample size was too small to run statistical analyses on the rates of CH_4_ production and oxidation in snag cores.

### Microbial Community Sampling and DNA Extraction

Deadwood was sampled using a protocol similar to Tláskal et al. [59]. A 1.27 cm drill bit was sterilized using ethanol and then used to drill a hole horizontally to the center of each snag at the midpoint of the location where the static flux chamber was installed. Three replicates were taken at each location. Shavings were collected aseptically using an ethanol-sterilized scoopula and deposited into a sterile 50 mL Falcon tube (Thermo Fisher Scientific, Waltham, MA). Samples were immediately placed on ice in the field and stored in a freezer until they were transported back to the lab. Upon receipt in the lab, samples were held at − 80 °C [60] before processing for DNA extraction.

Prior to DNA extractions, samples were ground independently using liquid nitrogen and a sterile mortar and pestle; this process was repeated three times per sample [40] to obtain a finely ground product. Genomic DNA was extracted from each sample following the protocol in the MP Biomedicals FastDNA Spin Kit for Soil (Santa Ana, CA). A NanoDrop 2000 Spectrophotometer (Thermo Fisher Scientific, Waltham, MA) was used to quantify DNA in each extraction. Samples were subsequently stored at − 15 °C before shipment to North Carolina State University’s Genomic Sciences Laboratory in Raleigh, NC, for Illumina sequencing.

### Microbial Sequence Analysis and Bioinformatics

Library construction and DNA sequencing were performed at North Carolina State University’s Genomic Sciences Laboratory. DNA sequencing was performed on an Illumina MiSeq platform (Illumina, San Diego, CA) using the V3/V4 region of the 16S rRNA gene. The 341F/805R primer pair, which targets bacteria and archaea [61], was selected from Klindworth et al. [62]. The sequence data from this study are archived in the NCBI Sequence Read Archive database under the BioProject number PRJNA994044.

Forward and reverse sequences were 300 bp long. The DADA (version 1.21.1) pipeline was used to generate an ASV table for the samples [63]. Initial steps in the pipeline included removing forward and reverse primers, filtering and trimming the reads, where the forward reads were trimmed to 275 bases and the reverse reads to 250 bases. The DADA2 algorithm inferred the sequence variants in each sample after learning the base pair transition error rates. The forward and reverse reads were merged to generate the denoised sequences, and merged sequences that were outside of the range of 383–433 bp were removed as potential non-target hits. Chimeric sequences were removed and taxa were assigned to the sequence variants using the Silva reference database (v. 132) [64, 65]. A phylogenetic tree was constructed using the R package *phangorn* [66], where the multiple sequence alignment was performed using the R package *DECIPHER* [67]. The tree construction was based on the steps in Callahan et al. [68].

Analyses were performed in R [69] with extensive use of the R package *Phyloseq* (version 1.30.0) [70]. The average sample abundance was 293,061. One sample replicate for T-4 had a low count (21,374) as well as a lower sequencing quality compared to the other samples and was thus removed. There were 12,021 taxa for the 29 remaining samples. The taxa were filtered such that the taxa Kingdom classification assignment was to either ‘Archaea’ or ‘Bacteria’. Taxa with a Family classification to ‘Mitochondria’ or an Order classification to ‘Chloroplast’ were also removed. Any ASVs that had fewer than 10 reads in total were removed. After the taxa filtering steps, 9,139 ASVs remained.

The relative abundance (RA) of the taxa was examined for the individual snag replicates on the Kingdom, Phylum, Class, Order, and Family levels. RA is a metric that represents the proportion of total reads and is measured on a scale of 0.0–1.0. The average RA was also calculated for the snag replicates where the RA values were averaged over the snag replicates for each taxonomical assignment. The taxonomical assignments that are ‘NA’ were excluded. The relative abundances were also examined for ASVs that were assigned to cultivated taxa with confirmed methanogenic and methanotrophic capacities (Table S1). The average RA for each ASV was calculated for the snag replicates. The ASVs were then assigned to the categories of *Methanogens*, *Methanotrophs*, or *Other*. The *Methanotrophs* category was replaced by three subcategories based on their abundance, where methanotroph ASVs with a RA ≤ 0.01 were assigned to category *Methanotroph_L1*, 0.01 < RA ≤ 0.02 were assigned to category *Methanotroph_L2*, and RA > 0.02 were assigned to category *Methanotroph_L3*. Methanogenic ASVs in the data included *Methanobacteriales* and *Methanomassiliicoccales*; whereas methanotrophic ASVs included *Methylocapsa*, *Methylocella*, and *Methylovirgula*. The relative abundance of the 56 ASVs that were assigned to taxonomic ranks known to contain methanotrophs (i.e., *Methylocapsa, Methylocella,* and *Methylovirgula*) were then summed for each snag, averaged over the snag replicates, and scaled to represent relative abundance within the methanotrophic community of each snag. A limitation of 16S rRNA data is that functions cannot be reliably known, especially for methane oxidation because the trait is polyphyletic. Therefore, all lineages identified herein as methanotrophs or methanogens are conservatively considered putative methane-oxidizers or -producers for the purpose of analysis.

Alpha diversity measures of the Shannon Index and the complement of Simpson’s Diversity (1-D) were calculated after the dataset was rarefied to 30,000 reads. There were 8,730 ASVs in the rarefied set. All additional analyses used the non-rarefied dataset. The beta diversity of the snag replicates was examined with principal component analysis (PCA) [71]. Initially, the ASV count data were transformed using the centered log-ratio (clr) transformation using the *CoDaSeq* R package (version 0.99.4) [72]. Before applying the transformation, any existing zero counts were replaced with non-zero values and this calculation was performed with the *zCompositions* R package (version 1.3.3.1) using the *count zero multiplicative* method [71]. The Euclidean distance matrix was calculated for the clr transformed ASV counts using the distance function in phyloseq. The ordinate function (method = “RDA”) in phyloseq was used to generate the PCA plot.

Hierarchical clustering was performed to visualize sample clustering in a method similar to that described by Gloor and Reid [73]. Initially, the ASV read counts were agglomerated on the class level. The zero value replacement and clr transformation steps were applied to the class level count table as described above. The distance matrix was calculated in R using the *dist* function (method = “euclidean”) and hierarchical clustering was performed with *hclust* (method = ”ward.D2″) to generate a dendrogram of the 29 snag samples.

## Results

### Snag-Atmosphere Methane Fluxes

Of the ten static flux chambers that were used to measure CH_4_ fluxes across the snag-atmosphere interface, five passed quality control standards (Table 1). Methane production was measured in three out of ten chambers, with an average flux of 0.21 ± 0.15 mg CH_4_ m^−2^ h^−1^. Snags 5, 8, and 9 had measured fluxes of 0.08, 0.51, and 0.03 mg CH_4_ m^−2^ h^−1^, respectively. Methane consumption was measured in two out of ten chambers, with an average flux of − 0.07 mg CH_4_ m^−2^ h^−1^. Snags 4 and 10 had measured fluxes of − 0.11 and − 0.03 mg CH_4_ m^−2^ h^−1^, respectively.

**Table 1:** Snag-atmosphere CH_4_ fluxes, CH_4_ sampling from trunk airspace, and potential CH_4_ production and oxidation incubations for each snag and height/incubation condition, where applicable

Snag	Snag-atmosphere CH_4_ Flux (mg CH_4_ m^−2^ h^−1^)		Trunk airspace CH_4_ (µL L^−1^)^a^			Potential methane production (ng CH_4_ g^−1^ h^−1^)		Potential methane oxidation (ng CH_4_ g^−1^ h^−1^)	
	Production	Consumption	30 cm	60 cm	120 cm	Anoxic	Oxic	Anoxic	Oxic
1	–	–	23.5	12.5	4.4	–	–	–	–
2	–	–	5.3	6.2	3.9	0.7	–	–	–
3	–	–	26.2	21.9	10.2	–	–	–	–
4	–	− 0.11	6.6	6.5	6.8	–	–	–	− 0.5
5	0.08	–	4.2	2.4	2.5	–	–	–	− 1.0
6	–	–	29.8	9.3	2.7	–	0.6	–	–
7	–	–	9.1	13.4	11.4	–	–	–	–
8	0.51	–	113.0	86.8	13.9	–	–	–	–
9	0.03	–	15.0	3.3	2.8	–	–	–	− 2.0
10	–	− 0.03	4.5	4.4	3.9	–	–	–	− 1.0
Mean ± SE	0.21 ± 0.15	− 0.07	23.7 ± 7.5	16.7 ± 5.3	6.3 ± 1.9	–	–	–	− 1.13 ± 0.31

^a^For reference, background atmospheric CH_4_ concentration was 6.3 ± 2.0 µL L^−1^. The unit µL L^−1^ is equivalent to ppm

*Note*: A dash (–) indicates that a sample did not meet quality control standards. Mean ± standard error are calculated where sample size is greater than two samples

### Methane Sampling from Trunk Airspace

There were no significant differences in CH_4_ concentrations in ambient air by height among the 30, 60, and 120 cm measurements, which is indicative of atmospheric mixing. The mean concentration of CH_4_ in trunk airspace at 30, 60, and 120 cm decreased by height (Table 1): 23.7 ± 7.5, 16.7 ± 5.3, and 6.3 ± 1.9 µL L^−1^, respectively. However, there were no significant differences among heights, possibly due to high variability in the dataset. Snag 7 was unique in that the trunk CH_4_ concentration at 60 cm was 1.5 × higher than at 30 cm, and the trunk CH_4_ concentration dropped 15% between 60 and 120 cm. This indicates a hot spot of CH_4_ in this snag at the 60 cm sample height, the location where the sample for trunk microbial community analysis was obtained. Trunk airspace CH_4_ concentrations were significantly elevated (P = 0.01) compared to the air immediately outside of the trunk (6.3 ± 2.0 µL L^−1^) at 30 cm (23.7 ± 7.5 µL L^−1^), but not at 60 or 120 cm (Table 1).

### Potential CH_4_ Production and Oxidation Incubations

One core from each snag was incubated under anoxic conditions to measure potential CH_4_ production, and one core from each snag was incubated under oxic conditions to measure potential CH_4_ oxidation. Of the 20 cores, 6 passed quality control standards (Table 1). A single core from snag 2 produced CH_4_ under anoxic conditions at a measured rate of 0.7 ng CH_4_ g^−1^ h^−1^. A single core from snag 6 produced CH_4_ under oxic conditions at a measured rate of 0.6 ng CH_4_ g^−1^ h^−1^. Four cores, taken from snags 4, 5, 9, and 10, showed evidence of CH_4_ oxidation under oxic conditions, with an average rate of − 1.13 ± 0.31 ng CH_4_ g^−1^ h^−1^ (range − 0.5 to − 2.0 ng CH_4_ g^−1^ h^−1^). Snag 9 had the strongest rate of CH_4_ oxidation at − 2.0 ng CH_4_ g^−1^ h^−1^.

### Trunk-Associated Microbial Communities in Snags

**Fig. 2:**
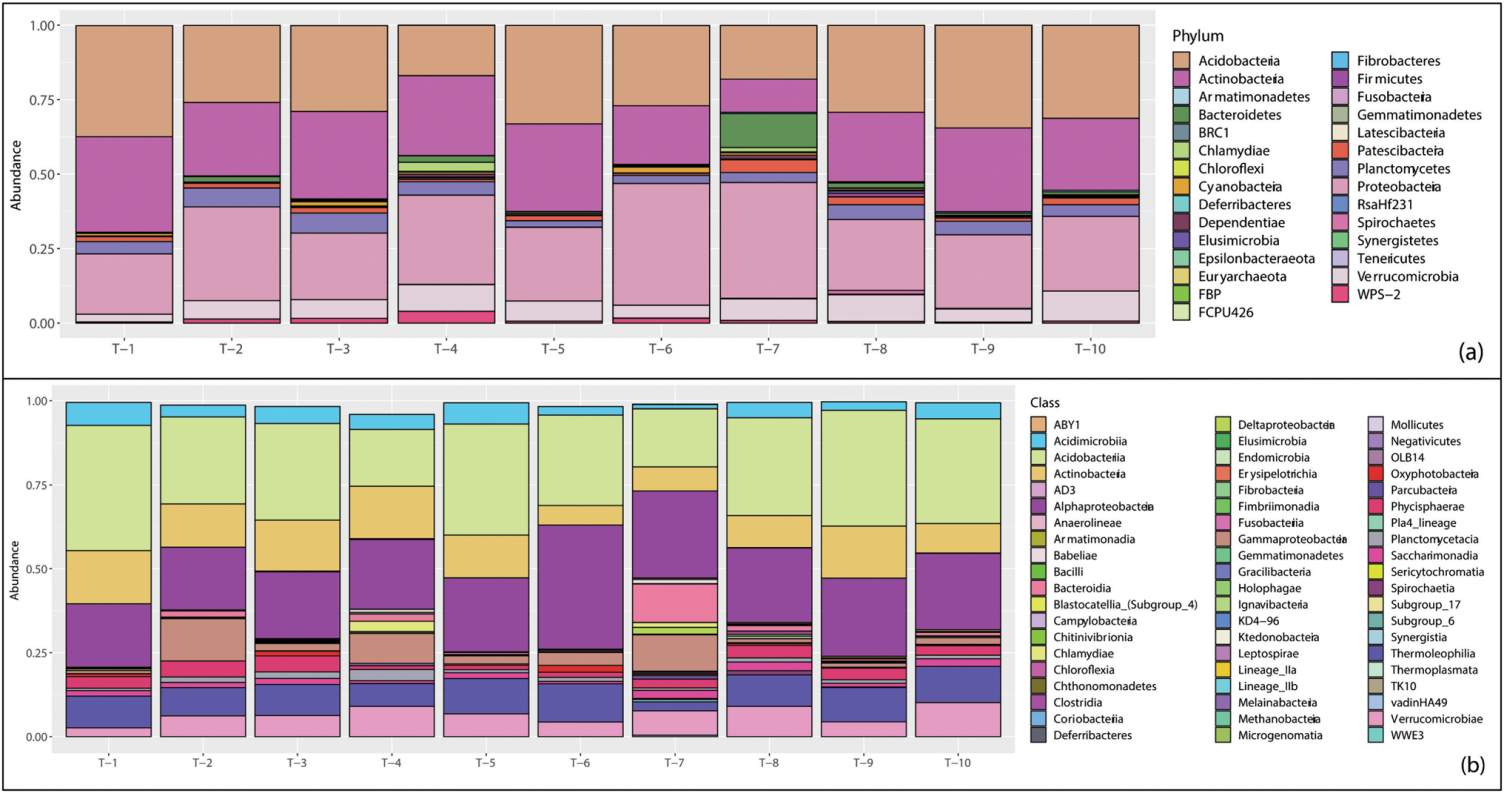
Average relative abundance of taxa in each snag at the level of (**a**) phylum and (**b**) class. Relative abundances were averaged over the snag replicates. Taxonomic assignments for ASVs that were not resolved (i.e., coded as *N/A*) were not included in the bar plots, which can result in total abundance values being less than 1

In nine of the ten snags (Fig. 2a), *Proteobacteria*, *Acidobacteria*, and *Actinobacteria* were the top three most abundant phyla, with mean relative abundances of 28%, 28%, and 26%, respectively. *Verrucomicrobia* and *Planctomycetes* were the fourth and fifth most abundant phyla, with mean relative abundances of 6% and 4%, respectively. Individual snags had absolute abundances of phyla that varied from the mean, but the pattern in mean relative abundance of phyla was consistent among 9 out of 10 snags. Snag 7 differed from this pattern in that the top five most abundant phyla were *Proteobacteria* (39%), *Acidobacteria* (18%), *Bacteroidetes* (11%), *Actinobacteria* (11%), and *Verrucomicrobia* (7%). Across all ten snags, the distribution of the relative abundance of all phyla was consistent among all snag replicates (Fig. S1).

In seven of the ten snags (Fig. 2b), *Acidobacteria, Alphaproteobacteria*, and *Actinobacteria* were the top three most abundant classes, with mean relative abundances of 28%, 23%, and 12%, respectively. Individual snags had absolute abundances of bacterial classes that varied from the mean, but the pattern in mean relative abundance of classes was consistent among 7 out of 10 snags. In snag 6, the top three most abundant classes were *Alphaproteobacteria* (37%), *Acidobacteria* (28%), and *Thermolephilia* (11%). In snag 7, the top three most abundant classes were *Alphaproteobacteria* (26%), *Acidobacteria* (17%), and *Bacteroidia* (11%). In snag 10, the top three most abundant classes were *Acidobacteria* (31%), *Alphaproteobacteria* (22%), and *Thermolephilia* (11%). Across all ten snags, the distribution of the relative abundance of classes of bacteria was consistent among all snag replicates (Fig. S2).

**Fig. 3:**
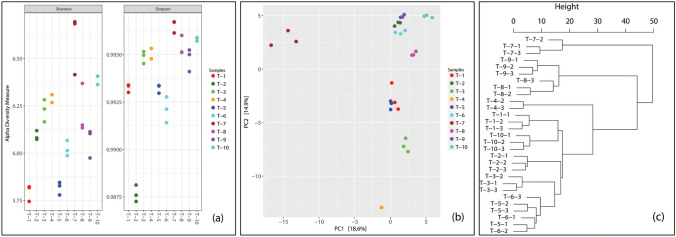
Alpha (**a**) and beta (**b**) diversity of microbial communities in standing dead trees in Gull Rock State Game Lands. Each snag replicate is indicated by an individual point in panels (**a**) and (**b**). Alpha diversity measures (**a**) were calculated after the dataset was rarefied to 30,000 reads; there were 8,730 ASVs in the rarefied set. Principal component analysis (**b**) was used to examine beta diversity. Hierarchical clustering was performed to generate a dendrogram of the 29 snag samples (**c**), identifying three clusters: a cluster solely represented by snag 7, a second cluster including snags 8 and 9, and a third cluster including all remaining snags

Snags contained diverse microbial communities (Fig. 3a), with no distinct similarities in community structure that were evident following PCA (Fig. 3b). Hierarchical clustering (Fig. 3c) revealed three distinct clusters of similar communities: a cluster solely represented by all three replicates from snag 7, a second cluster including the snag replicates from snags 8 and 9, and a third cluster including all remaining snags.

An analysis of the top 20 ASVs contained in each sample (Table 2, Supplementary File Traits Associated with Top 20 ASVs) revealed that nine of the ten snags had microbial communities that can be broadly characterized as acid-tolerant aerobes, with a number of species that have been previously documented in association with plants and/or have the capacity to degrade plant polymers (Table 3). Snag 7 was an outlier (Fig. 3) and had the most diverse microbial community of all snags, and it was the only snag with a ca. 1:1 ratio of aerobes:facultative aerobes/anaerobes among the top 20 ASVs. Eight of the top 20 ASVs in snag 7 had cultured representatives that are facultative anaerobes, microaerophiles, or facultative aerobes (Supplementary File Traits Associated with Top 20 ASVs). One of the top 20 ASVs in snag 7, *Telmatospirillum*, is a genus that has been isolated from a methanogenic enrichment culture (Table 3). In addition, several of the top 20 ASVs, including *Acidothermus*, *Granulicella*, and *Terracidiphilus*, are species that are capable of degrading plant polymers; *Endobacter* and *Myoavidus* have been isolated from plants (Table 3).

**Table 2:** Analysis of the proportion of genera representing the top 20 ASVs in each snag associated with functional traits relevant to the decomposition of deadwood. For a full analysis of the traits associated with the top 20 ASVs, please reference the supplementary file *Traits Associated with Top 20 ASVs*. On average, the top 20 ASVs represented 23 ± 2% of the total snag-associated microbial community (range, 7–29%)

Snag	Aerobes^a^ (%)	Facultative aerobes and facultative anaerobes^b^ (%)	Saprophytes^c^ (%)	Wood associated taxa^d^ (%)	Taxa capable of methanotrophy (%)
1	72.6	0	43.0	0	0
2	93.0	0	14.4	12.4	5.2
3	85.5	0	28.9	6.7	3.7
4	89.4	0	29.2	16.8	2.9
5	85.2	0	40.2	10.4	0
6	79.1	0	40.7	42.5	8.1
7	36.3	37.1	16.9	0	0
8	83.6	0	50.2	3.7	0
9	79.3	0	42.9	13.4	0
10	90.9	0	49.2	22.5	6.8
Mean ± SE	79.5 ± 5.2	3.7 ± 3.7	35.5 ± 4.0	12.8 ± 4.0	2.7 ± 1.0

^a^With the exception of snag 7, all remaining top 20 ASVs in each snag were unclassified at the level of genus; therefore, the remaining ca. 20% of the top 20 ASVs in each snag are unclassified with respect to aerobic vs. anaerobic lifestyles in this analysis

^b^There were no obligate anaerobes found in the top 20 ASVs of any snag

^c^Taxa classified as saprophytes are capable of using plant sugars as carbon sources and/or degrading plant polymers

^d^Wood-associated taxa may directly or indirectly contribute to deadwood decomposition

**Table 3:** Relevant traits associated with the top 20 ASVs found in decaying wood from standing dead trees at Gull Rock State Game Lands, Hyde County, NC. Semicolons in the reference list separate citations for taxa, which are listed in alphabetical order

Trait	Taxa	Reference
Acid tolerant	*Acidipila, Acidisoma, Acidisphaera,* *Acidocella, Acidothermus, Bryobacter,* *Bryocella, Edaphobacter, Granulicella* *Methylacidiphilae, Methylovirgula,* *Occallatibacter, Telmatospirillum*	Okamura et al. [103]; Belova et al. [104], Mieszkin et al. [105]; Hiraishi et al. [106]; Jones et al. [107], Kimoto et al. [108], Okamoto et al. [109]; Mohagheghi et al. [110]; Kulichevskaya et al. [111]; Dedysh et al. [112]; Belova et al. [113], Koch et al. [114], Wang et al. [115], Xia et al. [116]; Pankratov and Dedysh [117]; Dunfield et al. [118], Hou et al. [119], Islam et al. [120], Mohammadi et al. [121], Picone et al. [122]; Gwak et al. [91]; Foesel et al. [123]; Sizova et al. [76]
Capable of using plant sugars as carbon sources and/or degrading plant polymers	*Acidisoma, Bryobacter, Bryocella, Conexibacter, Granulicella, Humibacter,* *Terracidiphilus,*	Mieszkin et al. [105]; Kulichevskaya et al. [111]; Dedysh et al. [112]; Seki et al. [124]; Pankratov and Dedysh [117]; Choi et al. [125]; Garcia-Fraile et al. [126]
Co-cultured with methanogens	*Telmatospirillum*	Sizova et al. [76]
Co-cultured with methanotrophs	*Bryocella, Edaphobacter*	Dedysh et al. [112]; Koch et al. [114]
Endohyphal bacteria	*Mycoavidus*	Ohshima et al. [127]
Facultative aerobes	*Haoranjiania*	Zhang et al. [128]
Facultative anaerobes	*Haoranjiania, Hyphomicrobium, Sediminibacterium, Telmatospirillum*	Zhang et al. [128]; Martineau et al. [129], Xu et al. [130]; Kim et al. [131], Wu et al. [132]; Sizova et al. [76]
Methanotrophs	*Methylacidiphilae, Methylovirgula*	Dunfield et al. [118], Hou et al. [119], Islam et al. [120], Mohammadi et al. [121], Picone et al. [122]; Gwak et al. [91]
Methylotrophs	*Hyphomicrobium, Methylovirgula*	Izumi et al. [133], McDonald et al. [134], Urakami et al. [135]; Vorob’ev et al. [44]
Microaerophiles	*Mycoavidus*	Ohshima et al. [127]
Plant associated	*Acidisoma, Acidocella, Endobacter,* *Humibacter, Methylovirgula, Mycobacterium, Sphingomonas, Terriglobus,*	Mieszkin et al. [105]; Kimoto et al. [108]; Ramírez-Bahena et al. [136]; Kim et al. [137], Lin et al. [138]; Vorob’ev et al. [44]; Bouam et al. [139]; Huang et al. [140], Rivas et al. [141], Takeuchi et al. [142], Yan et al. [143]; Whang et al. [144]
Wood associated	*Acidisoma, Humibacter, Methylovirgula*	Mieszkin et al. [105]; Lin et al. [138]; Vorob’ev et al. [44]

### CH_4_-Cycling Communities in Snags

An analysis of the most abundant phyla in each snag revealed that the relative abundance of taxa that contain known methanogens was extremely low. If present, potential methanogens were represented by the archaeal orders *Methanobacteriales* (snags 8 and 9) and *Methanomassiliicoccales* (snags 8 and 9); there were no cyanobacterial lineages in the sequencing data that indicated an expanded capacity for methanogenesis beyond the *Archaea*. Eighty percent of the snags (snags 1–7 and 10) did not have any detectable putative methanogenic *Archaea*. In snags 8 and 9 putative methanogenic *Archaea* were not in the top 20 most abundant phyla for each snag, with relative abundances of 4.6 × 10^–5^ and 1.7 × 10^–5^, respectively. Overall, if methanogens were present, they represented a mean relative abundance of 3.2 × 10^–5^ within each snag.

Members of the genera *Methylocapsa, Methylocella, and Methylovirgula* were identified in snags (Table S1). Each of these taxa contain cultured representatives that demonstrate methanotrophic activity, though not all members from these lineages are obligate methanotrophs and/or capable of methanotrophy (e.g., [44, 74, 75]). *Methylocapsa aurea* is a facultative methanotroph that is capable of using other C1 compounds, such as methanol and formate, as well as the multicarbon compound acetate (C2) [75]. Members of the genus *Methylocella* are capable of using methanol (C1), as well as other multicarbon compounds such as acetate (C2), ethanol (C2), pyruvate (C3), malate (C4), and succinate (C4) [74]. *Methylovirgula ligni* are facultative methylotrophs, capable of utilizing other C1 compounds such as methanol, as well as the multicarbon compounds ethanol (C2), pyruvate (C3), and malate (C4) [44]. An analysis of the top 20 ASVs found in each snag (Supplementary File Traits Associated with Top 20 ASVs) revealed that snags 2, 3, 4, 6, and 10 contained taxa with cultivated representatives that are capable of methanotrophy (Table 2, Table 3) within the top 20 ASVs, all with relative abundances of 1–2%. *Methylovirgula* was found in snags 2, 3, 4, and 10, and was the second most abundant ASV in snag 6. *Brycoella* and *Edaphobacter*, genera that have been co-cultured with methanotrophs (Table 3 and citations therein), were identified among the top 20 ASVs in 5 out of 10 snags (Table 2). The methylotrophic genera *Hyphomicrobium* and *Methylovirgula* (Table 3 and citations therein) were found within the top 20 ASVs in 6 out of 10 snags. There were no putative methanogens identified in the top 20 ASVs in any of the snags (Supplementary File Traits Associated with Top 20 ASVs); however, the 16th most abundant ASV in snag 7, *Telmatospirillum* (Table 3) has been co-cultured with methanogens [76].

**Fig. 4:**
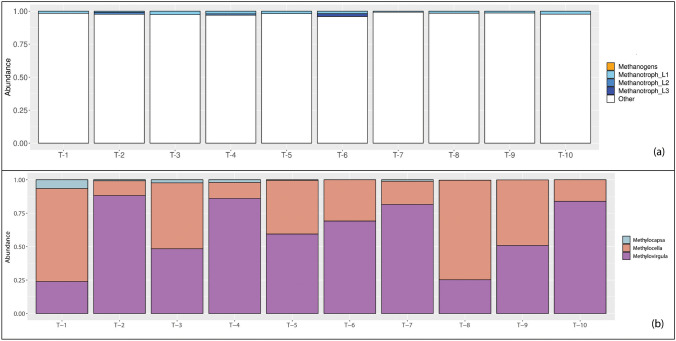
Diversity of taxa known to contain CH_4_-cycling microorganisms in each snag. (**a**) ASVs were assigned to the categories of *Methanogens*, *Methanotrophs*, and *Other*. Methanogens include *Methanobacteriales* and *Methanomassiliicoccales*. Methanotrophs include *Methylocapsa*, *Methylocella*, and *Methylovirgula*. Methanotrophs were divided into three subcategories based on their abundance, where methanotroph ASVs with a relative abundance ≤ 0.01 were assigned to category *Methanotroph_L1*, methanotrophs with a relative abundance 0.01 < x ≤ 0.02 were assigned to category *Methanotroph_L2*, and methanotrophs with a relative abundance > 0.02 were assigned to category *Methanotroph_L3*. All remaining ASVs, including putative uncultivated methanogenic and methanotrophic taxa, are categorized as *Other*. (**b**) Relative abundances of the 56 ASVs that were assigned to taxonomic ranks known to contain methanotrophs were summed for each snag, averaged over the snag replicates, and then scaled to represent a proportion of the methanotrophic community in each snag

**Fig. 5:**
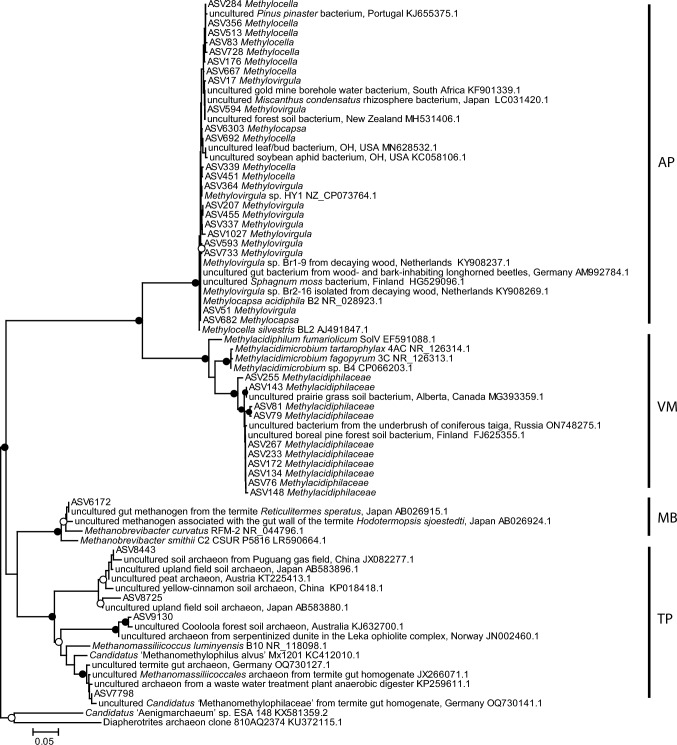
Neighbor-joining tree inferring the phylogenetic relationship between representative SSU rRNA sequences retrieved from snag cores that were also assigned to groups known to oxidize or produce methane (shown by ASV number and classification) and related cultured and uncultured sequences from GenBank. Order names are abbreviated as follows: *Methanobacteriales* (MB), *Methanomassiliicoccales* (MM), *Hyphomicrobiales* (HM), and *Methylacidiphilales* (MA). Nodes with bootstrap values between 80–100 that were also supported by maximum likelihood analysis are marked with filled circles and those with bootstrap values between 50–75 that were also supported by maximum likelihood analysis are marked with unfilled circles. There were no nodes with bootstrap values between 75–80 that were also supported by maximum likelihood. The scale bar indicates fractional differences in nucleotide sequences

Taxa associated with methanotrophy (Fig. 4) were identified in all of the snags, representing a range of 1–4% of all of the ASVs identified in each snag, with a mean relative abundance of 2% (Fig. 4a). In contrast, taxa associated with methanogenesis (Fig. 4a and Fig. 5) were represented by 2 ASVs that were identified in 2 of the 10 snags (snags 8 and 9), with a mean relative abundance in each snag of < 0.0001%. In snags where taxa associated with methanotrophy and methanogenesis were both present (i.e., snags 8 and 9), methanotrophs were, on average, two orders of magnitude more abundant than methanogens. A neighbor-joining tree inferring the phylogenetic relationship between representative SSU rRNA sequences retrieved from snag cores that were also assigned to groups that have cultivated representatives known to oxidize or produce methane can be seen in Fig. 5.

The genus *Methylovirgula* dominated the potential CH_4_-consuming community in each snag (Fig. 4b), representing 62% of the methanotrophic ASVs in each snag (range 24–88%). *Methylocella* were also identified in each snag, representing 37% of the putative methanotrophic ASVs (range 11–70%). ASVs assigned to *Methylocapsa* were identified 80% of the snags (snags 1–8), but with a much lower mean relative abundance (1%, range 0–7%).

## Discussion

Microbial communities have been shown to modulate CH_4_ fluxes from live trees in lowland forests [43, 46], yet very little is known about the role of these communities in modulating CH_4_ fluxes from snags in lowland forests. To our knowledge, this study reports the first high-throughput characterization of the trunk-associated microbial community in snags in a ghost forest. Our results indicate that the snag-associated community is diverse and indicative of oxidative decomposition of deadwood. When present, taxa associated with methanogens were found in very low relative abundance in snags. In contrast, taxa associated with methanotrophs were identified in all snags and represented the sole CH_4_-cycling community in a majority of the snags. These data show that methanotrophs dominate the putative CH_4_-cycling community inside snags. As such, the phyllosphere-associated microbial community in snags has the potential to attenuate CH_4_ emissions across the plant-atmosphere interface in ghost forests, as previously suggested using isotopic and concentration information [33, 47].

### Phyllosphere-Associated Microbial Communities and Microbial Decomposition of Deadwood

In this study, the top three most abundant phyla and the most abundant classes were similar in most snags, corroborating data from Moll et al. [6] who measured deadwood logs in an upland forest in the Nationalpark Hainich in Central Germany. Our results did reveal some differences in individual snags, which could be a result of differences in the stage of decay and/or successional state of the community [5, 7, 59]. An analysis of the top 20 ASVs in each snag revealed that snags contain diverse microbial communities whose structure was indicative of oxidative decomposition of deadwood. The single exception to this pattern was snag 7, which contained a unique, highly diverse community that was indicative of a community shift towards adaptation to lower O_2_ concentrations. This assertion is corroborated by data from trunk airspace CH_4_ sampling that provided evidence of a more reducing microenvironment within the snag that would be more conducive to anaerobic decay; although differences in the microbial community have also been shown to vary due to differences in in C:N ratio [77, 78], pH and water content [6, 59, 79], wood density [80], stage of decay or tree age [7, 59, 81], and/or the phyllosphere microbial community composition [8, 35, 80]. Hierarchical clustering analysis grouped all three replicates of snag 7 together as a single cluster. Replicates from snags 8 and 9, which were the only two snags with standing water at their base, formed a second, distinct cluster and may serve as a larger conduit for methane emissions (Table 1, [82]). The third cluster contained replicates from all of the remaining snags, which were located on raised hummocks. Taken together, these data support research from upland systems [37, 83] indicating that microenvironmental conditions, either within decaying wood or the immediate surrounding, influence microbial community structure in deadwood stocks.

### Snags as an Interface for Methane Emissions from Ghost Forests

Previous research has demonstrated that, although the spatial footprint of snags over the soil/sediment is small, greenhouse gas emissions from snags can substantially increase the total ecosystem CO_2equivalent_ [33]. Both methanogens and methanotrophs have been isolated from [41, 42, 44] and identified in metagenomic analyses [39, 40, 43] of the phyllosphere communities of live trees. Yet, much less is known about the presence and prevalence of CH_4_-cycling microorganisms in standing deadwood stocks such as snags [37].

In order for snags to act as either a source or sink for the atmospheric flux of CH_4_, two conditions must be met: (1) gas accumulation within the trunk airspace and (2) flux of the gas across the snag-atmosphere interface. The data provided evidence that CH_4_ accumulated within the trunk airspace of snags. With the exception of snag 7, mean CH_4_ concentrations within the trunk followed an expected pattern [21, 47, 84], decreasing by height, an observation that supports a soil-based origin of trunk CH_4_. After the CH_4_ is produced by methanogens in wetland soils/sediments, it diffuses into the trunk of a snag, where it has the potential to be oxidized by trunk-associated microbial communities or escape to the atmosphere across the snag-atmosphere interface. Interestingly, snag 7 was the only snag with bark intact up the trunk, which likely helped to hold moisture in and decreased gas exchange with the atmosphere, both conditions that would increase anoxic microsites within the trunk and influence microbial community structure.

The results of the core incubations and static flux measurements indicate that snags can be a source and sink of atmospheric CH_4_ fluxes and support recent findings in snags in similar systems [21, 33, 47]. Snag CH_4_ emissions in this study were well within the range recorded in static flux measurements across 83 snags in a similar study across five different ghost forests in the Albemarle Pamlico Peninsula [33]. The quantitative difference in emissions between this study and Martinez and Ardón’s 2021 study are likely a result of the high degree of spatiotemporal variability that has been noted in several studies of plant-atmosphere CH_4_ fluxes [21, 48, 85–87]. In contrast, a study of live tree stems in seasonally flooded Amazonian forests measured much higher values ranging from 10 to 270 mg CH_4_ m^−2^ h^−1^ [30], indicating that edaphic factors that influence the sediment redox environment may impact the magnitude and direction of plant-based CH_4_ emissions. Potential methane production incubations of snag cores in this study indicated that small amounts of CH_4_ may be produced under either oxic or anoxic conditions for a low proportion of snags. These values were lower than those in a similar study that demonstrated CH_4_ production in snags across ghost forests in the Albemarle Pamlico Peninsula [47]. Finally, methane consumption incubations in this study demonstrated methane consumption in a much greater proportion of snags, compared to those measured by Martinez et al. [47], although maximum rates measured were approximately equal.

### Microbial Communities in Snags May Modulate Methane Emissions from Ghost Forests

Our sequencing data show that taxa that contain known methanogens are a very small proportion of the total community. Though methanogenesis has historically been associated with members of the *Archaea*, the capacity for methane production has recently been identified in other domains [15, 16]. Our sequencing primers did not target fungi, so we cannot comment on the capacity for fungal methane production in our sequencing data. However, a single core from snag 6 showed detectable methane production under oxic conditions, indicating that fungal CH_4_-producers may be present in snag phyllosphere communities. None of the methane-producing cyanobacterial lineages identified by Bižić et al. [16] were identified in the dataset. Instead, the methanogens in the dataset were associated with the archaeal orders *Methanobacteriales* and *Methanomassiliicoccales*. The low methanogen abundance across all snags does not appear to be an artifact of sequencing and/or primer section, as the primers used in this study have been shown to provide moderate coverage for *Archaea* [61] and used to amplify *Archaea* in other studies [88–90].

In contrast, taxa that contain known methanotrophs were identified in every snag. The presence of methanotrophic populations in the trunks of snags has been suspected for years [46, 47], but was confirmed for the first time in this study. *Methylovirgula* [91] were detected in the top 20 ASVs in 50% of the snags. The methanotrophic genera *Methylocella* [92–94] and *Methylocapsa* [95, 96] were found in lower relative abundance in our dataset. *Methylocapsa* are known to contain high-affinity enzymes that are capable of CH_4_ oxidation at atmospheric concentrations [95]; therefore, despite their low relative abundance, they may have a substantial impact on CH_4_ consumption within snags. In this study, mean trunk airspace CH_4_ concentrations were ca. 3–4× elevated above background atmospheric CH_4_, indicating an abundant CH_4_ supply for high-affinity oxidation.

Metagenomic surveys reveal that the uncultivated diversity in methanotrophic populations is likely extensive [25, 26, 39, 97, 98] and that many of these uncultivated lineages fall within taxa of bacteria (e.g., *Alphaproteobacteria*, *Gammaproteobacteria*, and *Verrucomicrobia*) that are known to contain methanotrophs and were identified in a high relative abundance in snags in this study. Genes associated with methanotrophy can move via horizontal gene transfer [99–101]. Most recently, Al-Shayeb et al. [102] identified a novel mobile extrachromosomal genetic element in *Methanoperedens* that supercharges methane oxidation capacity. The process of horizontal gene transfer allows for expanded functional capacities beyond what is transmitted vertically to an organism and our sequencing approach would not capture this level of functional diversity. Therefore, it is likely that the relative abundance of methanotrophic populations in our study represents a conservative estimate and that our study has not revealed the full extent of methanotrophic capacity in the microbial communities associated with snags.

Our data indicate that the microenvironment at the sample height was likely oxic and well-mixed with the atmosphere, conditions that would favor oxidative decomposition of deadwood and the proliferation of methane-consuming populations of microorganisms. However, if our stand was located in standing water, where waterlogging of woody tissues could lead to more extensive anoxic microsites within snags [82], microbial community structure could shift to favor methanogenesis over methanotrophy. Therefore, more research is needed to more fully resolve the abiotic factors that influence microbial community structure in standing deadwood stocks like snags [37, 83], and thus control carbon dynamics [38] in ghost forest systems.

## Conclusions

Methanogens and methanotrophs have previously been identified as members of phyllosphere-associated microbial communities, and prior studies demonstrate that the relative abundance of the two functional guilds within individual trees has the capacity to influence ecosystem-level CH_4_ dynamics [39, 43]. In this study, we show that the balance of these guilds in snags is tipped in the favor of oxidative decomposition and CH_4_ consumption. These results support multiple lines of evidence from prior work at the site [33, 47] and suggest that trunk-associated microbial communities in snags may attenuate CH_4_ emissions across the plant-atmosphere interface.

Although characterization of community structure is a necessary first-step, broadening 16S rRNA-based surveys to include metagenomic surveys that target the *pmoA* and *amoA* functional genes could expand our knowledge of the presence and prevalence of uncultivated lineages of methanotrophs in these environments [25, 48] and provide insight into metabolically active members of the community [39]. In addition, employing a multi-tiered biogeochemical approach (e.g., Jeffrey et al. [43]) could better resolve the quantitative importance of snag-associated methanotrophic communities in attenuating methane emissions from ghost forest wetlands.

### Supplementary Information

Below is the link to the electronic supplementary material.

Supplementary file1 (DOCX 1903 KB)

Supplementary file2 (XLSX 42 KB)

## Data Availability

The Illumina sequence data underlying this article are available in GenBank under BioProject accession number PRJNA994044. Summarized data underlying this article are available in the article and in its online supplementary material. At the time of publication, the raw data the support this article are not publicly available from the corresponding author, but the data are available upon request.
