# Supplementary material for: Microbial Communities in Standing Dead Trees in Ghost Forests are Largely Aerobic, Saprophytic, and Methanotrophic
Source: Curr Microbiol. 2024 Jun 19;81(8):229. doi: 10.1007/s00284-024-03767-w (PMC11186919; doi:10.1007/s00284-024-03767-w)
Supplement: Supplementary file 1 — Supplementary file1 (DOCX 1903 KB) [file 284_2024_3767_MOESM1_ESM.docx]

**Microbial communities in standing dead trees in ghost forests are largely aerobic, saprophytic, and methanotrophic**

Mary Jane Carmichael^1^*, Melinda Martinez^2^, Suzanna L. Bräuer^3^, and Marcelo Ardón^4^

^1^Departments of Biology and Environmental Studies, Hollins University, Roanoke, VA, 24020, United States of America

^2^U. S. Geological Survey, Eastern Ecological Science Center, Laurel, MD, 20708, United States of America

^3^Department of Biology, Appalachian State University, Boone, NC, 28606, United States of America

^4^Department of Forestry and Environmental Resources, North Carolina State University, Raleigh, NC, United States of America

***Corresponding author contact information:**

Postal Address: 7916 Williamson Rd., Roanoke, VA, USA, 24020

Phone: (336) 830-4041

Email: carmichaelm@hollins.edu

**Disclaimer:** Any use of trade, firm, or product names is for descriptive purposes only and does not imply endorsement by the U.S. Government.


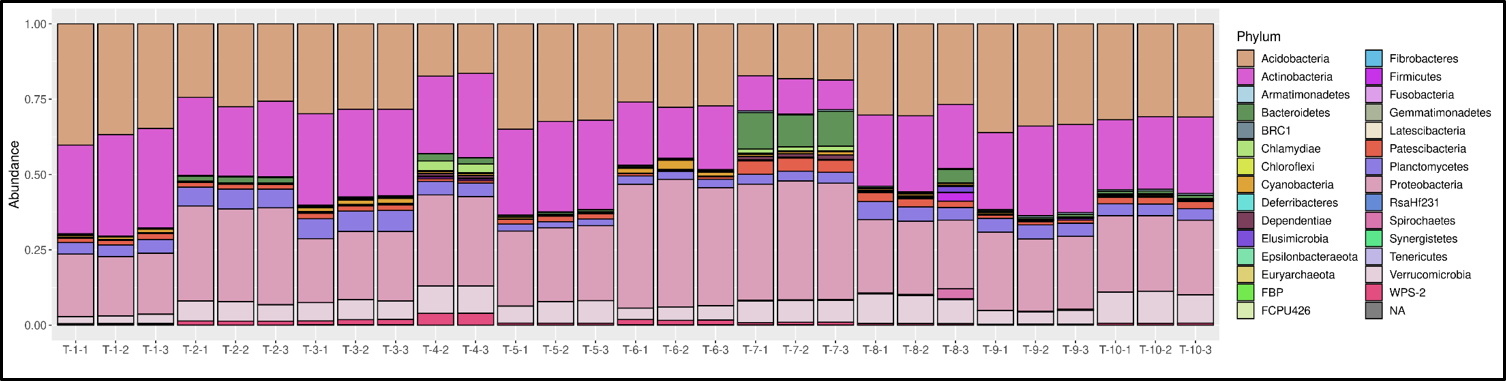


**Fig. S1** Relative abundance of phyla in each replicate. Samples are coded as T-tree number-replicate number


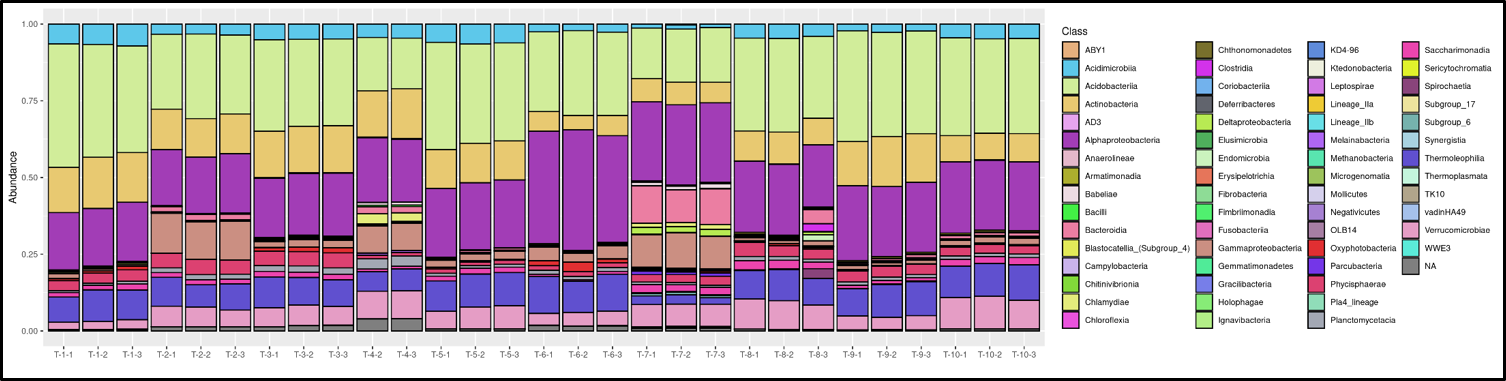


**Fig. S2** Relative abundance of classes in each replicate. Samples are coded as T-tree number-replicate number


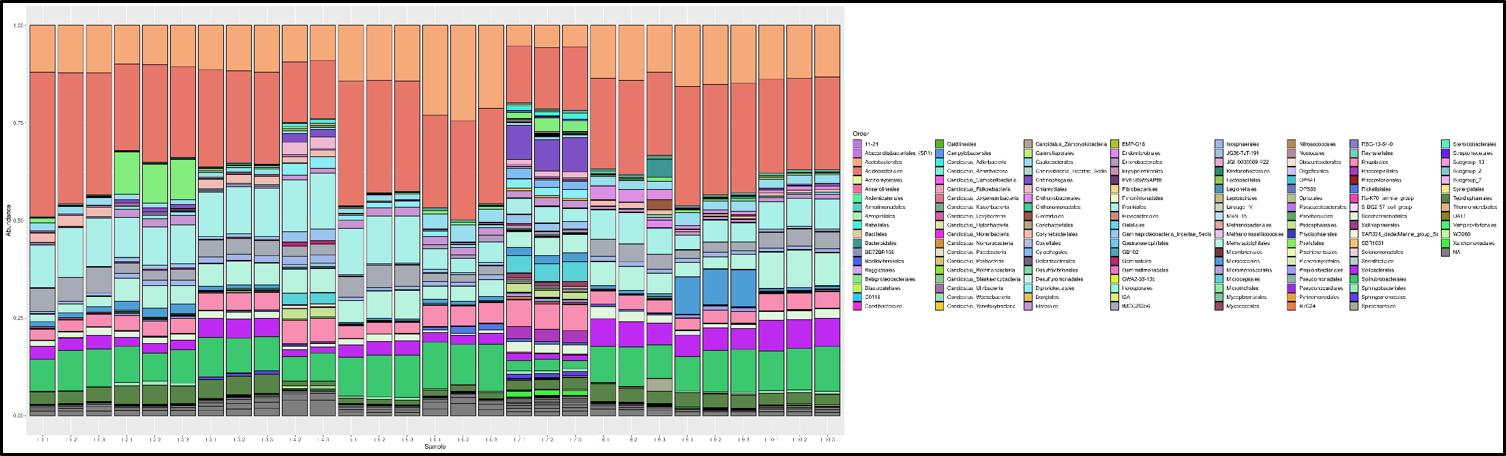


**Fig. S3** Relative abundance of orders in each replicate. Samples are coded as T-tree number-replicate number


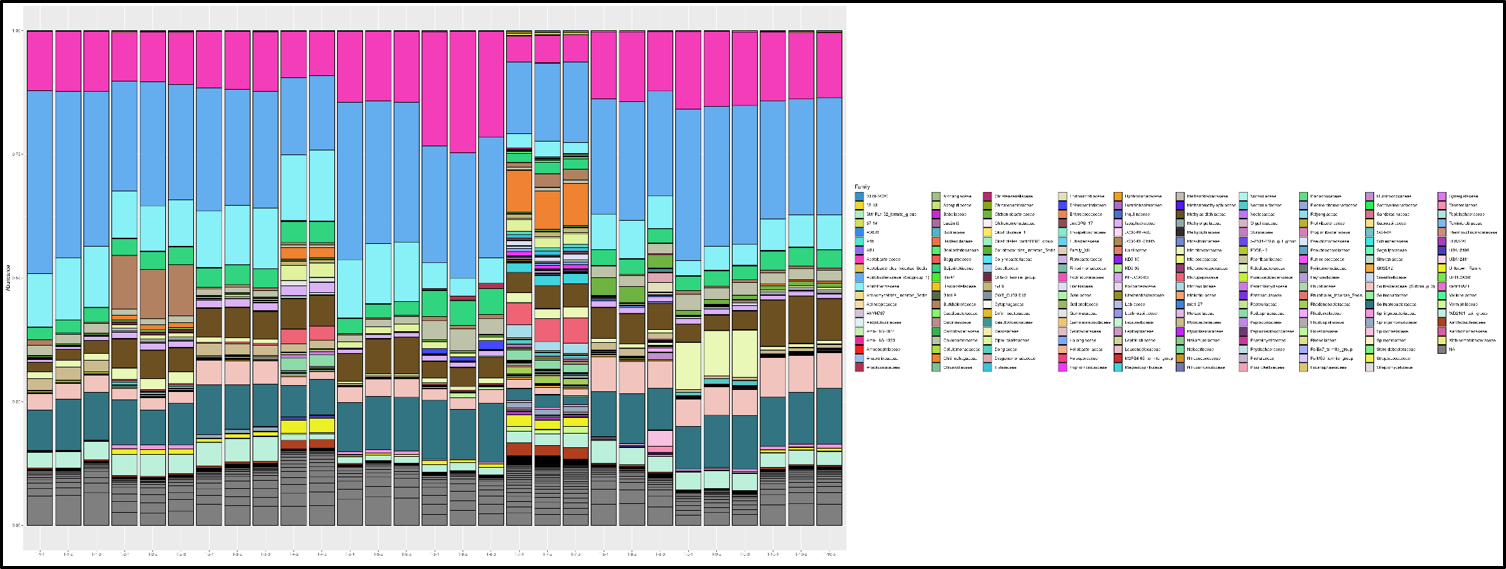


**Fig. S4** Relative abundance of families in each replicate. Samples are coded as T-tree number-replicate number

**Table S1** Taxonomic affiliations of cultivated and uncultivated methanotrophs. All listed genera have species that are capable of aerobic methanotrophy. Asterisks indicate clades that contain putative uncultivated methanotrophic lineages [1-4]. Putative uncultivated lineages have been identified through metagenomic surveys and by high-throughput sequencing of the particulate methane monooxygenase (*pmoA*) gene sequence

| **Domain** | **Phylum** | **Class** | **Order** | **Genus** |
| --- | --- | --- | --- | --- |
| Archaea | Euryarchaea | Methanomicrobia*^a^ |  |  |
| Bacteria | Gemmatimondotoa*^b^ |  |  |  |
|  | NC-10^*c^ | Methylomirabilia* | Methylomirabilales* |  |
|  | Proteobacteria | Alphaproteobacteria* | Hyphomicrobiales | *Methylocapsa, Methylocella, Methylocystis Methyloferula, Methyloceanibacter, Methylosinus, Methylovirgula* |
|  |  | Betaproteobacteria* |  |  |
|  |  | Gammaproteobacteria* | Methylococcales | *Methylobacter, Methylocaldum, Methylococcus, Methyloglobulus, Methylogoea, Methylohalobius, Methylomagnum, Methylomarinovum Methylomarinum, Methylomicrobium, Methylomonas, Methyloparacoccus, Methyloprofundus, Methylosarcina, Methylosoma, Methylosphaera,*  *Methylothermus, Methylotuvimicrobium, Methylovulum* |
|  | Verrucomicrobia* | Methyloacidophilum | [Verrucomicrobiota incertae sedis](https://www.ncbi.nlm.nih.gov/Taxonomy/Browser/wwwtax.cgi?mode=Undef&id=326457&lvl=3&lin=f&keep=1&srchmode=1&unlock) | *Methylacidimicrobium* |
|  |  |  | Methylacidiphilales | *Methylacidiphilum* |

^a^ The ANME-1, ANME-2, and ANME-3 clusters are known uncultivated lineages that group within the *Methanomicrobia*. The ANME-1 cluster is distantly related to *Methanosarcinales* and *Methanomicrobiales*, whereas the ANME-2 and ANME-3 clusters are members of the *Methanosarcinales*. The candidate genus *Methanoperedens* [5] is also a member of the *Methanomicrobia*.

^b^ The phylum *Gemmatimondotoa* contains *Candidatus* *Methylotropicum* *kingii* that is thought to be capable of aerobic methanotrophy [6].

^c^ NC-10 is a candidate phylum that contains *Candidatus Methylomirabilis oxyfera*, an organism that is thought to couple methane oxidation to nitrite reduction.

**References**

1. Knief C (2015) Diversity and habitat preferences of cultivated and uncultivated aerobic methanotrophic bacteria evaluated based on *pmoA* as a molecular marker. Frontiers in Microbiology 6:1346

2. Martins PD, de Jong A, Lenstra WK, van Helmond NAGM, Slomp CP, Jetten MSM, Welte CU, Rasigraf O (2021) Enrichment of novel *Verrucomicrobia*, *Bacteroidetes*, and *Krumholzibacteria* in an oxygen-limited methane- and iron-fed bioreactor inoculated with Bothnian Sea sediments. MicrobiologyOpen 10:e1175

3. Picone N, Blom P, Hogendoom C, Frank J, van Alen T, Pol A, Gagliano AL, Jetten MSM, D'Alessandro W, Quatrini P, Op den Camp HJM (2021) Metagenome assembled genome of a novel Verrocomicrobial methanotroph from Pantelleria Island. Frontiers in Microbiology 12:666929

4. Smith GJ, Wrighton KC (2019) Metagenomic approaches unearth methanotrophy phylogenetic and metabolic diversity. Current Issues in Molecular Biology 33:57-84

5. Al-Shayeb B, Schoelmerich MC, West-Roberts J, Valentin-Alvarado L, Sachdeva R, Mullen S, Crits-Christoph A, Wilkins MJ, Williams KH, Doudna JA, Banfield JF (2022) Borgs are giant genetic elements with potential to expand metabolic capacity. Nature 610:731-736

6. Bay SK, Dong X, Bradley JA, Leung PM, Grinter R, Jirapanjawat T, Arndt SK, Cook PLM, LaRowe DE, Nauer PA, Chiri E, Greening C (2021) Trace gas oxidizers are widespread and active members of soil microbial communitites. Nature Microbiology 6:246-256
